# Solution Structure of an Archaeal DNA Binding Protein with an Eukaryotic Zinc Finger Fold

**DOI:** 10.1371/journal.pone.0052908

**Published:** 2013-01-09

**Authors:** Florence Guillière, Chloé Danioux, Carole Jaubert, Nicole Desnoues, Muriel Delepierre, David Prangishvili, Guennadi Sezonov, J. Iñaki Guijarro

**Affiliations:** 1 Institut Pasteur, Unité de RMN des Biomolécules, Département de Biologie Structurale et Chimie, Paris, France; 2 CNRS UMR 3528, Paris, France; 3 Université Paris 7 Denis Diderot, Paris, France; 4 Institut Pasteur, Unité de Biologie Moléculaire du Gène chez les Extrêmophiles, Département de Microbiologie, Paris, France; 5 Université Pierre et Marie Curie, Paris, France; Spanish National Cancer Center, Spain

## Abstract

While the basal transcription machinery in archaea is eukaryal-like, transcription factors in archaea and their viruses are usually related to bacterial transcription factors. Nevertheless, some of these organisms show predicted classical zinc fingers motifs of the C2H2 type, which are almost exclusively found in proteins of eukaryotes and most often associated with transcription regulators. In this work, we focused on the protein AFV1p06 from the hyperthermophilic archaeal virus AFV1. The sequence of the protein consists of the classical eukaryotic C2H2 motif with the fourth histidine coordinating zinc missing, as well as of N- and C-terminal extensions. We showed that the protein AFV1p06 binds zinc and solved its solution structure by NMR. AFV1p06 displays a zinc finger fold with a novel structure extension and disordered N- and C-termini. Structure calculations show that a glutamic acid residue that coordinates zinc replaces the fourth histidine of the C2H2 motif. Electromobility gel shift assays indicate that the protein binds to DNA with different affinities depending on the DNA sequence. AFV1p06 is the first experimentally characterised archaeal zinc finger protein with a DNA binding activity. The AFV1p06 protein family has homologues in diverse viruses of hyperthermophilic archaea. A phylogenetic analysis points out a common origin of archaeal and eukaryotic C2H2 zinc fingers.

## Introduction

It is now well established that transcription in archaea, one of the three domains of life, displays characteristics of both eukaryal and bacterial transcription [1], [2]. The minimal basal machinery in archaea consists of an RNA polymerase and the general transcription factors TBP (TATA-box-binding protein) and TFB (transcription factor B), required for transcription initiation. These proteins are homologues of the eukaryal RNA polymerase II (RNAPII), TBP and TFIIB proteins, respectively. In particular, the eukaryal and archaeal RNA polymerases show a striking structural similarity [3], [4]. The archaeal basal machinery is thus homologous structurally and functionally to the core components of the eukaryal RNAPII machinery. In contrast, non-general transcription factors (TF) in archaea are often bacterial-like, and only a few are predicted to be of eukaryal type [1], [2]. For instance, a recent *in silico* analysis based on 52 archaeal genomes suggested that over 50% of the predicted transcription factors show at least one homologue in bacteria, about 43% are specific to archaea and less than 2% have homologues in eukaryotic organisms [5]. Though some transcription factors in archaea have been analysed in detail [6]–[8], transcription regulation in archaea is still poorly documented.

The presence in archaea of proteins with predicted zinc finger domains of the C2H2 or C2HC type is intriguing as the so-called “classical” zinc finger, hereafter named ZNF, is considered to be an eukaryal-specific motif. Initially discovered in the transcription factor TFIIIA from *Xenopus* oocytes [9], the ZNF domain has been shown to be very abundant in eukaryotes (*e.g.* 3% of human genes encode ZNF-containing proteins), practically absent in bacteria with some exceptions as in plant pathogens [10] and scarce, but represented in archaea and their viruses. The classical ZNF motif consists of a short (∼30 residue-long) sequence that uses two or three cysteines and two or one histidines to coordinate a zinc ion (C2H2 or C2HC types, respectively). The ZNF domains fold into a characteristic structure consisting of an α-helix and a β-hairpin held together by the zinc ion and hydrophobic interactions between hydrophobic residues at conserved positions of the sequence. Most of the proteins containing ZNF domains that have been characterised are involved in transcription regulation and bind DNA through their ZNF domains, although ZNF domains can also mediate protein-RNA or protein-protein interactions [11], [12]. ZNFs bind to DNA by inserting the α-helix into the major groove and use three or four exposed residues of the helix to make specific contacts with three or four DNA bases [13], [14]. To recognise their target DNA in a cellular context, ZNFs are usually present in tandem repeats separated by a short linker. Each ZNF repeat binds specifically to DNA using the α-helix and the repeats wrap around the DNA. Some ZNFs like SW15, ADR1 or GAGA, however, are present in only one to three copies and use extensions of the ZNF motif to further contact DNA [15]–[17].

Hyperthermophilic archaea that thrive in hot springs (>80°C) are infected by viruses that show unique morphological and genomic properties that distinguish them from bacteriophages and eukaryal viruses [18]. The majority of the proteins of these viruses does not have detectable homologues in the databases, however, a relatively high proportion is predicted to carry transcription-factor associated folds (up to ∼10% of proteins encoded in genomes with about 50 putative genes) [19]. The abundance of putative TFs in the genomes of hyperthermophilic archaeal viruses probably reflects the importance of transcription regulation in the life cycle of the viruses. As in the case of their hosts, the majority of the predicted TFs are bacterial-like and display a ribbon-helix-helix (RHH) or a helix-turn-helix (HTH) fold. One viral predicted TF, the SvtR protein from virus SIRV1, has been characterised and shown, indeed, to display a RHH structure and to repress transcription of viral genes [20]. Structural analysis of another viral protein (E73) coded by the SSV-like virus SSV-RH, also revealed the presence of a RHH motif involved in DNA recognition [21]. In addition to bacterial-like TFs, archaeal viruses from the *Rudiviridae*, *Lipothrixviridae*, *Fuselloviridae* and the *Bicaudaviridae* families as well as the unclassified viruses STSV1 and STIV typically present one or two sequences with ZNF motifs.

In this work, we focused on the protein AFV1p06 coded by the gene *gp06* of the virus AFV1 (*Acidianus* filamentous virus 1 [22]), which infects the hyperthermophilic crenarchaeon *Acidianus hospitalis*. The protein has 59 residues and displays a single ZNF motif with the second zinc-binding histidine of the motif missing. The ZNF motif (28 residues) is flanked by N- and C-terminal regions of unknown structure. AFV1p06 has homologues in crenarchaeal spindle-shaped viruses from the *Fuselloviridae* family (SSV1, SSV2, SSV4, SSV5, SSV6 and SSVK-1), and is distantly related to eukaryal ZNF containing proteins [19]. Here, we describe the solution structure of AFV1p06 and analyse its DNA binding capabilities.

## Materials and Methods

### Cloning, Protein Expression and Purification

The gene *AFV1p06* of AFV1 (NC_005830.1, also called ORF59a) was amplified by PCR using primers AFV1p06NdeI (5′-ATGCCATATGATTGAGGTTTCTAGTATGG-3′) and AFV1p06XhoI (5′-ATTTCTCGAGTCAGATAATCTTGTTTACAT-3′). The PCR product was digested with *NdeI* and *XhoI* and ligated with *NdeI* and *XhoI* digested pET-30a (Novagen) plasmid vector.

Recombinant AFV1p06 was expressed without any tag or cloning-derived additional residues using *Escherichia coli* Rosetta™ (BL21 DE3) pLysS (Novagen) cells. Cultures at 37°C in rich (Luria-Bertani broth) or in minimal M9 media for ^15^N or ^15^N/^13^C labelling, induction with 1 mM isopropyl-β-thio-galactopyranoside, cell harvesting after four hours of induction and cell freezing at −80°C were performed as described [20].

AFV1p06 was purified from inclusion bodies. Frozen cells were thawed, suspended in 50 mM HEPES pH 7.4, 150 mM NaCl, 3 mM dithiothreitol (DTT, buffer A) and lysed with a French press at 4°C adding phenyl-methane-sulphonyl fluoride. The cell lysate was centrifuged 20 min at 7000 g and 4°C and the supernatant was discarded. The cell pellet was suspended in buffer A supplemented with DNAse and RNAse to eliminate nucleic acids and centrifuged at 7000 g for 20 min at 4°C. The cell pellet was then suspended in buffer A containing 1% Triton to eliminate hydrophobic compounds, centrifuged and washed twice with buffer A by means of suspension and centrifugation cycles. The washed pellet was solubilised in buffer A containing 6 M urea (buffer B) and loaded into a size exclusion chromatography column (Sephacryl HR100, GE Healthcare) pre-equilibrated with buffer B. The sample was eluted with buffer B and the AFV1p06 containing fractions were pooled and dialysed at low concentration (∼25 µM) and temperature (4°C) against buffer A containing 500 mM arginine and 50 µM ZnCl_2_ (buffer C) to renature the unfolded protein. After renaturation, arginine was eliminated by dialysis against buffer C prepared without arginine (buffer D) and loaded on an ion-exchange column (SP Sepharose, GE Healthcare) previously equilibrated with the loading buffer. Proteins were eluted using a linear gradient of NaCl from 150 mM to 1 M in buffer D. The AFV1p06 containing fractions, which eluted at *ca.* 650 mM NaCl, were pooled, dialysed against the desired buffer (typically 50 mM HEPES pH 7.4, 100 or 150 mM NaCl, 50 µM ZnCl_2_, 3 mM DTT) and concentrated by centrifugation using Vivaspin (Sartorius) tubes with a 3 kDa cut-off. Protein preparations were aliquoted and kept at −80°C or used directly for NMR experiments.

Protein preparations were homogeneous as assessed by SDS-PAGE and NMR; protein integrity and identity were checked by SELDI-TOF mass spectrometry (Jacques d'Alayer, Microsequencing Facilities, Institut Pasteur). The concentration of the protein was determined using a molar extinction coefficient of 5960 M^−1^.cm^−1^ calculated from its sequence [23].

### Flame Atomic Emission Spectrophotometry

Experiments were carried out at the Ecole Polytechnique (Palaiseau, France) on a Varian AA220 spectrophotometer equipped with an air-acetylene burner. Readings were performed at 213.9 nm in the peak height mode. Two samples in buffer A were analysed: one was prepared as described above and extensively dialysed to eliminate free zinc ions from the sample; the second one was obtained without adding ZnCl_2_ during renaturation or the following purification steps and adding a forty fold excess of NaEDTA relative to the protein during the refolding step.

### Oligonucleotides

Oligonucleotides were purchased from Proligo (Sigma-Aldrich). Double-stranded DNA was obtained by annealing the corresponding single strand oligonucleotides following standard techniques. For PAGE experiments, oligonucleotides were ^32^P radiolabelled using the T4-polynucleotide kinase (Fermentas).

### DNA Binding

Two 25-bp duplex DNA oligonucleotides, called dsATcomb (top strand sequence 5′-AATGATTCTAAGTATCTTAGAAACA-3′) and dsGCcomb (top strand sequence 5′-AGGGTGGCAGCGTCGGAGCCTCGCA-3′) were obtained by annealing the corresponding single strand complementary oligonucleotides. Prior to annealing, one strand of each oligonucleotide was ^32^P-radiolabelled. Each double-stranded labelled oligonucleotide (75 nM) was incubated with increasing amounts of AFV1p06 (from 0 to 2 µM) for 15 min at 48°C in 20 µl of binding buffer: 50 mM HEPES, 10 µM ZnCl2, 150 mM NaCl, 5% (v/v) glycerol, 0.02% Tween, 3 mM DTT, pH 7.4. The binding buffer was supplemented with 50 ng/µL of unspecific salmon sperm DNA. The DNA-protein mixtures were deposited in a non-denaturing 6% 37.5:1 acrylamide/bisacrylamide gel. PAGE was run in TBE buffer (89 mM Tris-borate, 2 mM NaEDTA, pH 8.3). After migration, the gel was vacuum-dried, exposed with Amersham Biosciences Hyperfilm™ MP and developed with a Kodak X-OMAT 2000 processor.

Binding of dsATcomb and dsGCcomb to AFV1p06 was also tested by competition experiments in which labelled dsATcomb or dsGCcomb at a fixed concentration (75 nM) were used as probes in electromobility gel shift assays (EMSA) and unlabelled dsGCcomb or dsATcomb at varying concentrations (0, 0.5, 1, 2 and 5-fold molar ratio of unlabelled/labelled oligonucleotide), were used as competitors. Oligonucleotides and AFV1p06 (0.5 µM) were incubated 15 min at 48°C in 20 µL of binding buffer. PAGE, gel drying and development were performed as described above.

### NMR Samples

Samples were prepared in buffer E: 50 mM HEPES pH 7.4, 100 mM NaCl, 44 µM ZnCl_2_, 4.5 mM DTT, 12% D_2_O. Protein concentration typically ranged between 0.4 and 1.0 mM for ^15^N labelled and ^13^C/^15^N labelled samples.

### NMR

Experiments were performed on a Varian NMR System 600 spectrometer (Agilent Technologies, Santa Clara) with a proton resonating frequency of 599.4 MHz. The spectrometer was equipped with a cryogenic probe. Spectra were recorded at 25°C and referenced to sodium 4,4-dimethyl-4-silapentane-1-sulphonate following IUPAC recommendations. Data were collected using VnmrJ 2.3A (Agilent Technologies), processed with NMRPipe [24] and analysed with NMRView 5.2.2 [25].

Standard two- and three-dimensional experiments were recorded to assign chemical shifts to the protein ^1^H, ^13^C and ^15^N nuclei: ^13^C or ^15^N HSQC (heteronuclear single quantum coherence) [26]), HNCO, HNCACB, CBCA(CO)NH [27], H(CC-TOCSY)NNH, C(CC-TOCSY)NNH [28], [29], (HB)CB(CGCD)HD and (HB)CB(CGCDCE)HE [30].

AFV1p06 backbone dynamics analysis was based on ^15^N relaxation experiments [31] used to calculate the longitudinal (R_1_) and transverse ^15^N (R_2_) relaxation rates.

*NMR and structure calculations–* Distance constraints for structure calculations were obtained from 3D ^13^C-edited (aromatic and aliphatic regions) and ^15^N-edited NOESY-HSQC (nuclear Overhauser effect spectroscopy - HSQC) experiments recorded with 120 ms mixing times [26], [32]. Proton J_HN-HA_ scalar couplings were calculated from a HNHA experiment [33], [34] and transformed into dihedral φ angle constraints as follows: −120°±25° for ^3^J_HN-Hα_ ≥ 8.0 Hz, −65°±25° for ^3^J_HN-Hα_ ≤ 5.5 Hz. Further dihedral φ and ψ constraints were obtained with Talos [35]. A backbone hydrogen bond in regions of secondary structure was added as distance constraint if the chemical shift data, the nOe pattern and the amide hydrogen exchange data were in agreement with a hydrogen bond, and if it was present in at least 75% of the structures calculated without any hydrogen bond. Hydrogen exchange was analysed using the HET-SOFAST experiment [36]: two spectra with (saturation) or without (reference) inversion of the water signal were acquired to evaluate the protection against exchange from the saturation transfer between water and amide protons. The residues with a ratio of intensities higher than 0.75 between the saturation and reference experiments were considered to be exchange protected.

NOESY spectra assignments and structure calculations were performed with ARIA 2.2 [37], [38] coupled to CNS 1.2 [39] following ARIA's standard protocols with spin diffusion correction.

Spin diffusion was corrected using an isotropic rotation correlation time of 6.3 ns (±0.6 ns), which was determined from ^15^N relaxation data as described in [40]. Chemical shift tolerances were set to 0.03 and 0.04 ppm for protons in the direct and indirect dimensions, respectively, 0.5 ppm for ^13^C and 0.35 ppm for ^15^N. For structure calculations and nOe (nuclear Overhauser enhancement) assignments, the zinc atom was coordinated with a tetrahedral geometry by the Sγ atoms of cysteines 13 and 16 and the Nε2 atom of histidine 29 (see Results section). Histine was unprotonated. The zinc ion was attached to the Sγ atom of residue 16, and the tetrahedral geometry was maintained by modifying the force field topology and parameter files. Once the nOes were assigned and the distance constraints were obtained, two different final structure ensembles were calculated using either the full-length protein (residues 1–59) or only the structured region (residues 7–51). The final structures were obtained by calculating 200 structures with ARIA 2.2/CNS 1.2 and refining the lower energy 150 structures in explicit water using the PARALLHDG 5.3 force field [41]. The 10 lowest-total-energy structures were selected. The quality of the structures was analysed with Procheck 3.5.4 [42], What_check [43], Molmol 2K.2 [44] and Pymol (Schrödinger LLC).

### Phylogenetic Analysis

To gather the amino acid sequences for phylogenetic analysis, we searched the non-redundant protein sequence database (nr) at NCBI for homologues of AFV1 virus p06-ORF59a (GI: 82056192) using the PSI-BLAST algorithm 2.2.26+ [45] in ten iterative steps with default parameters. Whenever the algorithm ran out of new proteins to include in the iteration, the protein with the best E-value and with a conserved C2H2 motif was manually picked. Sequences were aligned using the CLC Sequence Viewer software (CLC Bio, Denmark) with default parameters. The tree was calculated using the Neighbour-Joining method and a 100 replicate bootstrap analysis.

The protein knowledgebase UniProtKB database was questioned using the query “zinc AND finger AND C2H2” to obtain the sequences of proteins with predicted ZNF motifs in the three domains of life.

### Accession Codes

The structure and chemical shifts of AFV1p06 have been deposited in the PDB protein data bank (http://www.pdb.org) and the BMRB database (http://www.bmrb.wisc.edu) under the accession numbers 2LVH and 18570, respectively.

## Results

### Zinc Chelation by AFVIp06

As the homology of AFV1p06 to C2H2 and C2HC zinc fingers suggested that the protein could bind zinc, we performed flame photometry experiments on samples that had been carefully depleted of free zinc. These experiments confirmed that AFV1p06 binds zinc and showed that one mole of protein binds one mole of zinc. In addition, sedimentation-diffusion equilibrium ultracentrifugation experiments performed at a 50 µM concentration (Bertrand Raynal, Plate-forme de Biophysique, Institut Pasteur), and NMR ^1^H-^15^N HSQC spectra, which were invariant for AFV1p06 concentrations between 50 µM and 1.0 mM, indicated that the protein is monomeric up to millimolar concentrations. Thus, one AFV1p06 monomer binds one zinc ion.

In ZNF proteins, zinc is tetra-coordinated by two Cys and two His residues (C2H2) or three Cys and one His residue (C2HC). AFV1p06 contains two Cys residues (C13 and C16) and a single His residue (H29) that are part of the ZNF motif and that could be involved in zinc coordination. Nevertheless, the protein lacks the fourth zinc ligand, which could be either a water molecule or the side chain of residue E34 that in the sequence alignments with ZNF proteins is positioned close to the fourth zinc ligand (H or C). We performed a NMR chemical shift analysis to verify if residues C13, C16 and H29 could be involved in metal chelation. On the one hand, the Cβ and Cα chemical shifts of residues C13 and C16 were in agreement with those of metalloproteases [46], indicating that both cysteine Sγ atoms bind zinc; on the other hand, the comparison of the aromatic ring carbon chemical shifts (Cδ2 and Cε1) of H29 with that of histidine residues (deposited in the BMRB database) that bind or do not bind zinc, indicated that H29 binds zinc and that it ligates zinc through its Nε2 atom. This analysis was corroborated by a recently published method to determine the coordination of zinc by His residues based on the difference of the aromatic Cδ2 and Cε1 chemical shifts [47]: in the case of AFV1p06, this difference is 12.96 ppm, which corresponds well to the value observed for Nε2 coordination 12.32±0.86 ppm. Based on this experimental data, we calculated AFV1p06 structures considering that zinc was coordinated by residues C13 (Sγ), C16(Sγ) and H29 (Nε2) and we used the structures to determine the fourth ligand of zinc. Importantly, no bias that could influence the determination of the fourth ligand was introduced in the calculations because the nOe assignments for distance constraints were performed automatically.

### Resonance Assignments of AFV1p06

The ^1^H, ^15^N and ^13^C resonance frequencies of most backbone and side chain atoms were assigned (92%). Missing assignments mainly corresponded to exchangeable protons of lysine, arginine, asparagine and glutamine side chains, as well as to the backbone amide protons of residues S6, M7 and K23 (the assigned ^15^N-^1^H HSQC spectrum of AFV1p06 is shown in Figure S1).

### Structure of AFV1p06

**Figure 1:**
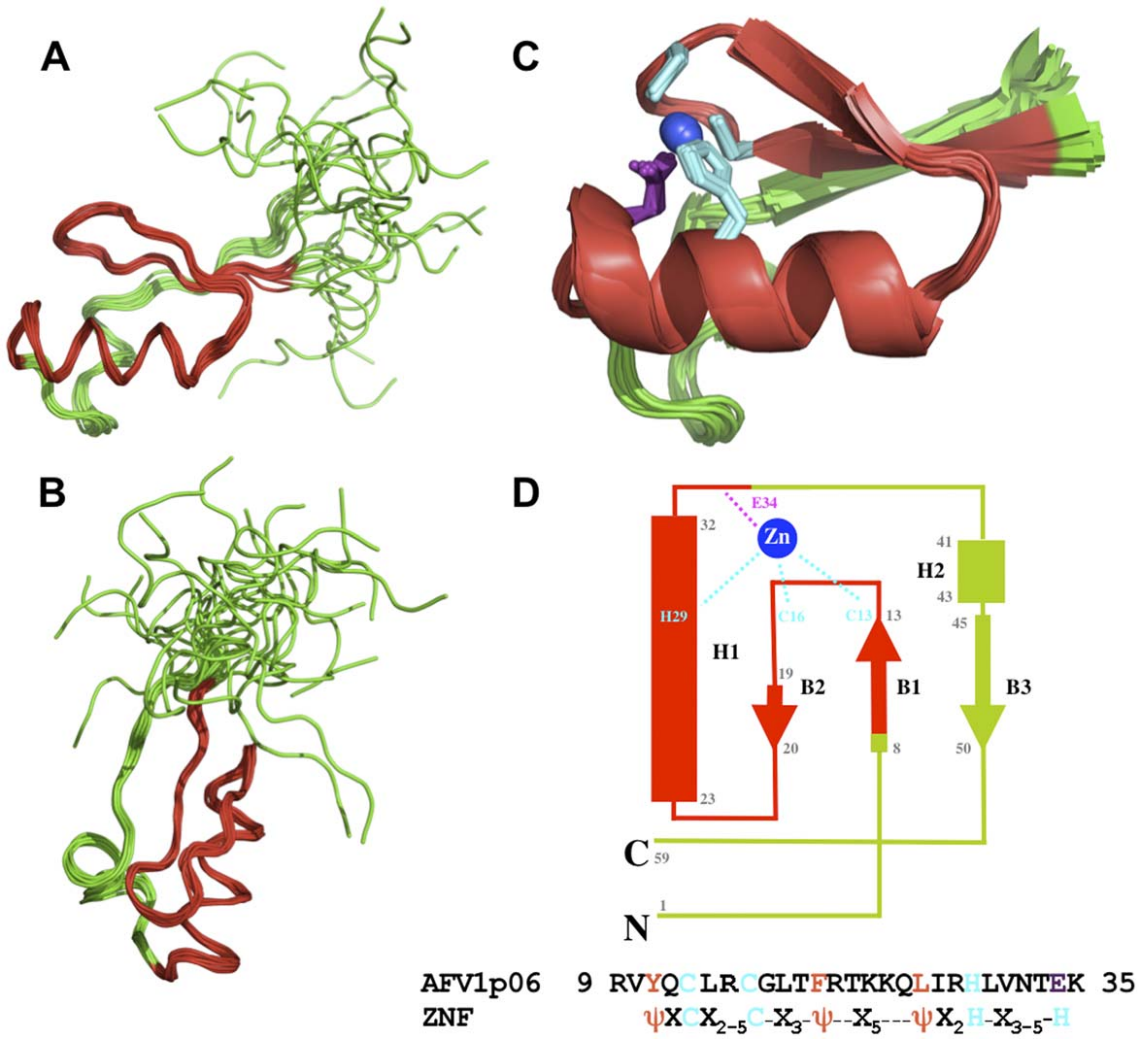
Structure of AFV1p06. The backbone superposition of the 10 structures calculated for the full-length protein is shown in two different orientations (A and B) and on a main-chain cartoon representation for residues 7–51 (C). A topology diagram of the structure, the sequence of AFV1p06 in the ZNF region (residues 9–35) and the ZNF sequence motif are shown in (D). Residues in the ZNF region are coloured in red. In (C), the side-chains of the residues that coordinate zinc are displayed in cyan (C13, C16 and H29) or violet (E34) and the zinc atom in blue. In (D), ψ stands for a hydrophobic residue. Helices are represented by rectangles and β-strands by arrows.

The structure ensemble of AFV1p06 shows a compact and convergent region between residues 8–50 and disordered N (1–7) and C (51–59) termini (Figure 1, Table 1). The structure consists of a three-stranded antiparallel β-sheet (residues 8–13, 19–20 and 45–50) packed against an α-helix (23–32), as well as of a short 3_10_ helix (41–43) located at the end of a long loop between the α-helix and the third strand of the β-sheet. As expected, the region with the ZNF sequence motif (residues 9–35) shows a typical zing finger fold with an antiparallel β-hairpin packed against an α-helix and with the zinc ion sandwiched between the latter structural elements. Indeed, a search for structural homologues in the DALI database (http://www.dali.server.org) with the structure of AFV1p06 between residues 9–35 produces over 150 ZNF structures with statistically significant scores and low root mean square deviations (RMSD≤1.8 Å over ∼25 CA atoms). When the coordinates of the structured region between residues 8 and 50 were used to find structural homologues, only the ZNF region gave significant hits, indicating that AFV1p06 shows a novel extension of the ZNF fold (loop with a 3_10_ helix+3^rd^ strand of ZNF β-sheet).

**Table 1:** Statistics for the ensemble of 10 structures calculated for AFV1p06 calculated with residues 7–51.

Constraints (residues 7–51)		Energies (kcal/mol)	
Unambiguous restraints	826	Total	−1682±21
Ambiguous distance restraints	133	Van der Waals	−170±13
Total number of distance restraints^a^	959	Electrostatic	−1867±35
Intra-residue | j−i | = 0	380	Mean of pairwise RMSD (Å) (8–50)^b^	
Sequential | j−i | = 1	202	Backbone atoms N, Ca, C′	0.60±0.13
Medium range 2≤| j−i |≤4	157	Heavy atoms	1.69±0.20
Long range | j−i |>4	220	Ensemble Ramachandran plot (8–50)^b^	
Backbone dihedral φ angle restraints	40	Residues in most favoured regions	90.8%
Backbone dihedral ψ angle restraints	37	additionally allowed	9.2%
Total backbone dihedral angle restraints	77		
Total number of hydrogen bonds	19	Structure Z scores (8–50)^b^	
Residual distance constraint violations		Second generation packing quality	−0.46±0.44
Number ≥0.3 Å	6	Ramachandran plot appearance	−1.62±0.70
Number ≥0.1 Å	68	Chi1/Chi2 rotamer normality	−2.38±0.96
RMS deviation from nOes (Å)^c^	0.0194±0.0037	Backbone conformation	−7.29±3.30
Residual dihedral angle constraint violations		Unsatisfied H-bond donors per molecule^b^	3.9
Number ≥5.0°	1	Unsatisfied H-bond acceptors per molecule^b^	0
RMS deviation from dihedrals (°)	0.512±0.136	Bumps (8–50)^b^	0

a Distance constraints used for structure calculations, which excluded fixed intra-residue distances.

b Values for the structured region (between residues 8 and 50).

c Includes nOe and hydrogen bond data.

The lack of convergence observed for the N- and C-termini of the protein correlates with a very low number of nOes shown by residues 1–7 and 51–59 and more specifically, with the absence of medium or long range nOes. This disorder is due to the dynamics of the protein as assessed by the ^15^N relaxation characteristics of the backbone amide groups. For instance, most of the N and C-termini amide groups showed low ^15^N transverse relaxation rates (R_2_) values relative to those observed for the rest of the protein, indicating high amplitude motions in the nanosecond-picosecond time scale (Figure S2). Also, residue S5 showed a very high R_2_ rate, and amide resonances of residues S6 and M7 were not observed, presumably due to exchange broadening (high R_2_ rates), suggesting that the latter residues exchange between different conformations in the microsecond-millisecond time scale. Thus, the N- and C-termini of AFV1p06 are highly dynamic.

The fourth ligand of the zinc ion was identified using the structure ensemble of AFV1p06: in all the structures, a side-chain oxygen atom of the carboxylic group of residue E34 is close to the zinc ion at a distance (1.99±0.04 Å) that is in agreement with those observed for zinc coordinated by a glutamic acid residue [1.95±0.08 Å, [48]]. This observation indicates that residue E34 is the fourth residue implicated in zinc coordination. Although a glutamic acid residue is not commonly observed as a zinc ligand, it coordinates zinc in some proteins in which the latter ion plays a structural role [48]. In AFV1p06, the zinc ion is tightly bound. Indeed, the protein retains zinc in the presence of a 10 fold excess of NaEDTA, as evidenced by the lack of changes in the NMR ^1^H-^15^N HSQC spectrum of the protein in the presence of the latter chelating agent.

### AFV1p06 Binds Preferentially to GC Rich DNA

The structure and zinc binding properties of AFV1p06 indicate that this protein is a classical zinc finger. As most of the ZNF proteins that have been characterised have been shown to bind double stranded DNA [14], we tested the DNA binding capabilities of AFV1p06. Because the putative binding site for AFV1p06 was not known, we initially performed EMSA experiments in the presence of unspecific DNA and high concentrations of the protein. We chose two DNA oligonucleotides that were extremely different in their nucleotide composition: the oligonucleotides, either single (ssDNA) or double stranded (dsDNA), were 24 nt long and exclusively composed of the succession of AT (polyAT) or CG (polyCG) pairs. The EMSA experiments indicated that AFV1p06 could bind dsDNA at micromolar concentrations and did not bind the corresponding single strand DNAs, and this independently of their DNA composition. Interestingly, the dsDNA polyCG oligonucleotide was clearly better recognised by AFV1p06 than the polyAT one (not shown). At high salt concentration (500 mM), AFV1p06 was also able to bind dsDNA and recognised better the polyCG oligonucleotide, suggesting that the interaction of this protein with DNA is not only based on protein – DNA-backbone electrostatic interactions but involves DNA bases.

Following these observations and in order to better characterise the DNA binding activity of AFV1p06, we designed two additional double strand oligonucleotides of 25 bp called “dsATcomb” (5′-AATGATTCTAAGTATCTTAGAAACA-3′) and “dsGCcomb” (5′-AGGGTGGCAGCGTCGGAGCCTCGCA-3′). The composition of these oligonucleotides was inspired by the crystal structure of the complex of the DNA-binding domain of the transcription factor Zif268 and its binding site. In the latter complex, each of the three ZNFs of Zif268 establishes specific contacts with 3 bases on one strand of the DNA [49]–[51]. Because the protein AFV1p06 has a single ZNF domain we hypothesized that its α-helix would interact with a short 3 nt DNA core site. The oligonucleotides “dsATcomb” and “dsGCcomb” were hence designed to carry regularly interspaced repetitions of different combinations of triplets (6 from 8 possible) composed of either A or/and T for the “dsATcomb” and G or/and C for the dsGCcomb oligonucleotides. With this combinatorial approach we tried to create one or several short DNA sub-regions in the analysed oligonucleotides that could be better recognised by AFV1p06 to test if the protein is able to discriminate between different DNA sequences.

**Figure 2:**
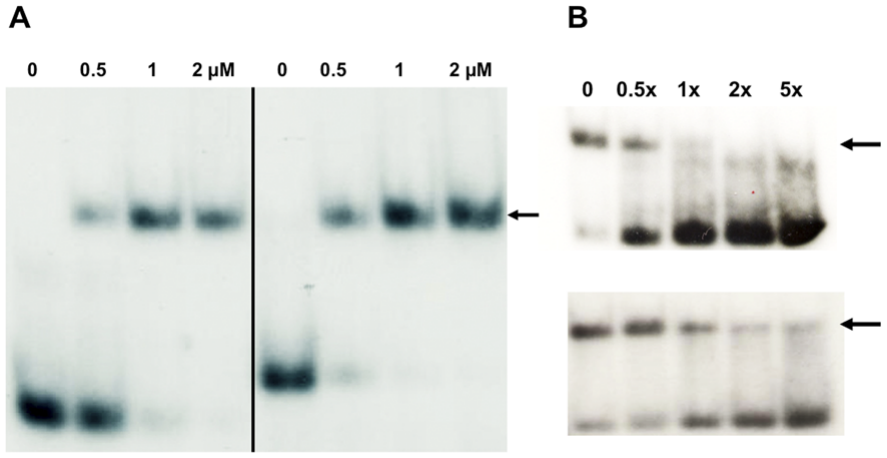
DNA binding of AFV1p06 monitored by PAGE-EMSA. (A) Binding to dsATcomb (left) and dsGCcomb (right) at a fixed concentration (75 nM) with increasing concentrations of AFV1p06 (0 to 2 µM). (B) Competition assays: experiments were performed in the presence of 0.5 µM AFV1p06 using “hot” radiolabelled dsATcomb and increasing amounts of dsGCcomb as a “cold” competitor (top), or radiolabelled dsGCcomb and increasing amounts dsATcomb as a “cold” competitor (bottom). The ratios between “hot” and “cold” oligonucleotides are indicated. Arrows show the position of the shifted DNA band.

PAGE-EMSA experiments were performed with radioactively labelled dsATcomb and dsGCcomb in the presence of an excess of non-specific unlabelled dsDNA (Figure 2A). Both oligonucleotides show a retard in migration in the presence of AFV1p06, indicating that the protein binds dsDNA on the µM concentration range. The binding of AFV1p06 to the GC-rich dsDNA oligonucleotide is at least twice more efficient than that observed for the AT-rich one. We also compared the efficiency of retardation of each oligonucleotide in the presence of the second one as a “cold” competitor. Even at a 1∶0.5 ratio between ^32^P labelled dsATcomb and unlabelled dsGCcomb a clear decrease of the signal corresponding to the shifted form of dsATcomb is observed, indicating that dsGCcomb can efficiently displace dsATcomb (Figure 2B). To observe a similar efficiency, a five-fold excess of “cold” dsATcomb has to be added to “hot” dsGCcomb. These results suggest that AFV1p06 shows a preference for GC motifs and thus can sense different bases and display some specificity in dsDNA recognition.

In an attempt to identify its presumed DNA target sequence, we followed a target candidate approach testing the binding of the protein to the region of its own promoter, as very often transcription regulators show *cis*-regulation. However, even if the promoter region of the *gp06* gene is unusually GC rich compared to the generally low GC content of the AFV1 genome (36%), under the *in vitro* conditions used, the efficiency of AFV1p06 binding to this region was not significantly different from that of a “non-specific” AT rich DNA from a non related virus (data not shown).

### Phylogenetic Studies

The AFV1p06-related proteins identified by the PSI-BLAST approach are divided into two clearly separated clusters of archaeal and eukaryal proteins that show a common origin (Figure S3). The archaeal proteins are grouped into two sub-clusters representing the two major archaeal phyla, *Cren*- and *Euryarchaeota*. No homologue could be identified in the domain of bacteria or in the third phylum of archaea, the *Thaumarchaeota*.

**Figure 3:**
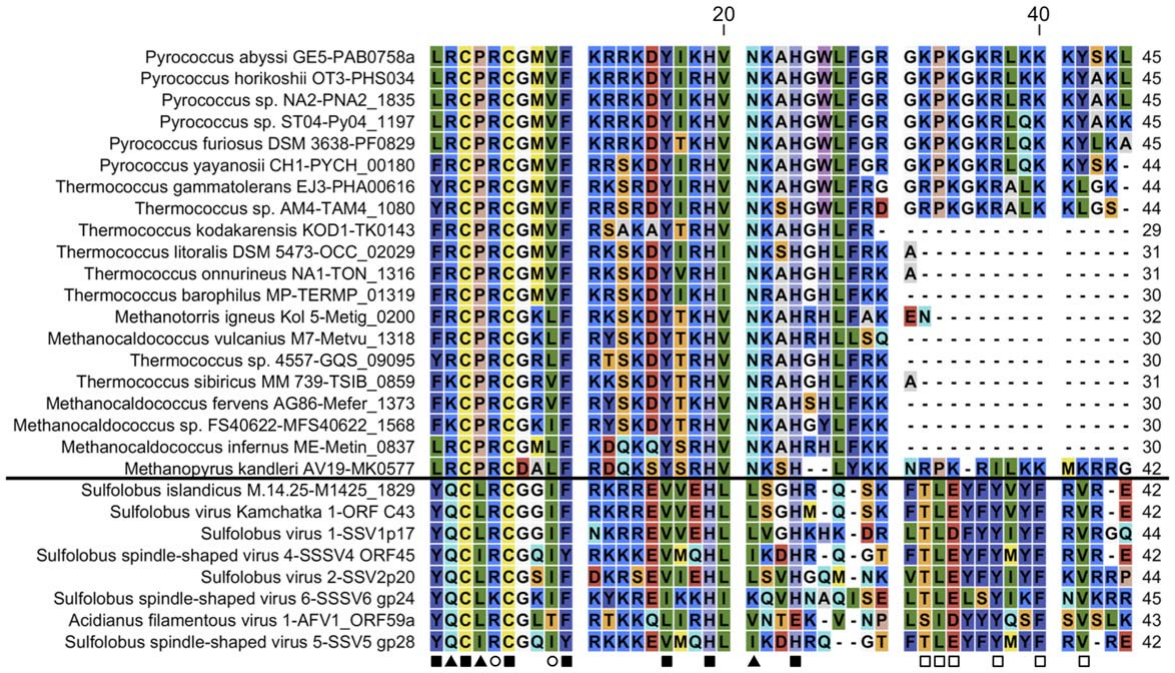
The AFV1p06 family of ZNF proteins in archaea. The figure shows the alignment of 27 hits corresponding to archaeal zinc finger proteins bearing an AFV1p06-like motif. Squares: position of the seven idiosyncratic residues of the ZNF fold; open circles: amino acids conserved in archaea but not in eukaryotes; triangles: amino acids specific to cren- or euryarchaea; open squares: amino acids conserved only in crenarchaea in the ZNF fold extension observed in AFV1p06 (loop+helix+3^rd^ strand of the β-sheet). The horizontal line separates the archaeal viral and cellular proteins.

The group affiliated to *Crenarchaeota* includes 8 representatives forming the “AFV1p06 family”. All of them are coded either by crenarchaeal viruses (SVS-K1 [52], SSV1 [53], SSV2 ([54], SSV4 and SSV5 [55], SSV6 [56] and AFV1 [22] or by proviruses integrated into the chromosome. In the case of *S. islandicus* M.14.25 the AFV1p06 homologue (M1425–1829) is annotated as being coded by a chromosomal gene but a more detailed analysis of this region, which shows typical viral *att*-like sites, clearly indicates the viral origin of the locus. The alignment of the predicted ZNF regions of these proteins indicates the presence of the seven highly conserved amino acids of the ZNF motif (two Cys, two His as well as three hydrophobic amino acids indicated by squares in Figure 3) except in the case of AFV1p06 in which the last His residue is replaced by a Glu residue. Thirteen additional amino acids (Figure 3) are well conserved in crenarchaeal C2H2-like proteins and six of them (indicated by asterisks) are localised in the loop, helix and third β-strand situated downstream to the ZNF fold. The latter six residues are exposed to the solvent in the structure of AFV1p06, suggesting that these residues are conserved because of their functional importance rather than their role in structure maintenance. The conservation pattern of the proteins in the AFV1p06 family and the nature of the amino residues, strongly suggest that the crenarchaeal C2H2-like proteins show the same structure extension of the ZNF fold as AFV1p06.

A distant group of putative ZNF proteins found in the *Euryarchaeota* (20 representatives) is very similar to the crenarchaeal viral AFV1p06 family in the ZNF motif region but does not show any conservation in the ZNF downstream extension. The origin, cellular or viral, of the genes belonging to this sister of the AFV1p06 group of euryarchaeal ZNF proteins is unclear.

Noteworthy, although AFV1p06 is the only protein in the alignment shown in Figure 3 that displays a Glu residue at the position of the second histidine of the C2H2 motif, it should be mentioned that in eukaryotic ZNFs, the 4^th^ ligand in the motif is also not conserved in a number of variant ZNFs. Conservation of a histidine seems thus less important at the fourth position, an observation that could be explained by the fact the 4^th^ ligand is not crucial to retain zinc binding capabilities as shown in a mutation/folding and stability analysis [57] or by the fact that it can be replaced by a water molecule [58].

## Discussion

The results described in this paper indicate that the archaeal virus protein AFV1p06 has a classical ZNF structure composed of an α-helix and a β-hairpin, a novel extension to this fold and disordered N and C terminal ends. In addition, the EMSA experiments show that the protein can bind to DNA at sub-micromolar concentrations and discriminate between different DNA sequences. Although the presumed biological target(s) of AFV1p06 on the AFV1 virus and or its host (*Acidianus sp.*) genome remains unknown, these results suggest that AFV1p06 could potentially be a transcription regulator.

**Figure 4:**
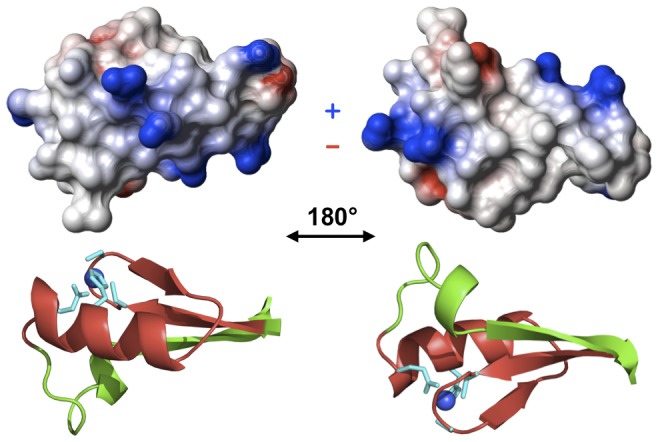
Surface electrostatic potential (top) and main-chain cartoon representations (bottom) of the structure of AFV1p06. A representative conformer was used. Positive charges are represented in blue and negative ones in red. The left and right views are rotated by 180° on the x-axis. The side chains of the residues that coordinate zinc are shown in cyan and the zinc ion as a blue sphere.

Classical zinc fingers usually bind to DNA using exposed residues at positions −1, +2, +3 and +6 of the α-helix that make specific contacts with DNA bases and establish other non-specific contacts with DNA as well. The electrostatic potential of AFV1p06 on the α-helix face is positive (Figure 4) and seems thus favourable for interacting with the negatively charged DNA poly-anion. Moreover, residues that occupy the DNA-contacting positions in AFV1p06 [T (−1), K (+2), Q (+3) and L (+6)] have been observed in ZNF–DNA complexes and could in principle establish specific contacts with DNA bases [13]. Manual docking of AFV1p06 into ZNF–DNA complex structures [PDB codes 2JPA and 2GLI, [59], [60]] suggests that AFV1p06 may also interact with DNA using the α-helix: the superimposition of the structure of AFV1p06 with that of ZNFs in complex with DNA indicates that residues at key positions of the helix could indeed make contacts with DNA and that the basic residues R8, R21 (−2) and K23 (+1), would be close to the DNA phosphate groups.

To recognise its cognate DNA in a cellular context, more than three or four specific DNA nucleotide bases/α -helix residue protein contacts must be established. To this end, eukaryal TFs usually show tandem repeats of ZNF motifs, or like in the case of the GAGA protein, make use of another module that also binds specifically to DNA [16]. The manual docking of AFV1p06 shows that the novel extension of the ZNF motif (the loop and third strand of the β-sheet) is far from the ZNF–DNA contact region (not shown), suggesting that this extension cannot directly contribute to the interaction without major conformational changes. Hence, two possibilities can be envisioned for specific binding of AFV1p06 to DNA: (i) the N- and/or C- disordered termini could participate in the interaction; (ii) another DNA binding protein could bind to AFV1p06 on the β-sheet face. Indeed, the hydrophobic character of the exposed β-sheet face and the conservation of its hydrophobicity in AFV1p06 homologues in SSV crenarchaeal viruses, make this face of the protein a good candidate for protein-protein interactions. In this respect, it is interesting to note that Pérez-Rueda and Janga have observed that although bacterial-like, predicted TFs in archaea are statistically smaller (shorter sequence) than in bacteria and specific ligand-binding modules are under-represented [5]. These authors have suggested that protein-protein interactions in archaeal TFs could mediate regulatory feedback. Similarly, it can be hypothesised that archaeal ZNF proteins could also need a protein partner for specific DNA recognition. In this sense, predicted archaeal and archaeal virus ZNFs appear in only one (∼74%) or two copies (∼20%) per protein in relatively short proteins (most often less than 100 residues). This situation is very rare in eukaryotes, in which very often ZNFs are present in tandem repeats. Although we cannot exclude that the ZNF fold in archaea may be preferentially used for protein-protein interactions or RNA-protein interactions, the fact that AFV1p06, which only contains one ZNF motif, does interact with non-specific DNA with relatively high affinities *in vitro* suggests to us that at least some of these proteins may be TFs and may use either other modules within the same protein or may interact with other proteins to control gene expression. Despite its small size, in the case of the N–terminal GATA-1 ZNF and its FOG ZNF partner, it has been observed that the ZNF fold can cope with simultaneous specific protein–DNA and protein–protein interactions or that two different ZNFs can bind to form heterodimers that bind DNA specifically [61]–[63]. Also, fungal GATA proteins involved in gene regulation display only one ZNF motif, bind to specific DNA sequences and can mediate protein–protein interactions that are important to regulate gene expression [64].

It should be underlined that proteins bearing the C2H2 zinc finger motif are essentially known and characterised in eukaryotes. In this domain of life, ZNFs are predicted to be coded by more than 1% of the genes compared to 0.07% for the archaea (278 examples in the Uniprot database) and only 0.003% (489 examples) for the bacteria. The phylogenetic analysis described here, clearly indicated a common origin of the AFV1p06-like ZNF domain for archaea and eukaryotes, and its absence in bacteria. In crenarchaea all the known genes have a virus related origin.

AFV1p06 is the first archaeal protein with an eukaryal ZNF fold to be characterised experimentally and the first for which the DNA binding and sequence preference capabilities have been demonstrated. It would be interesting in the future to identify its presumed targets on the AFV1 and/or *Acidianus* genomes and understand its role in the virus infection cycle.

## Supporting Information

Figure S1 **Assigned ^1^H-^15^N HSQC spectrum of AFV1p06.**
(PDF)

Figure S2 **Structural (number of nOe-derived distance constraints and structure backbone RMSD) and dynamics (amide ^15^N transverse relaxation rate) data of AFV1p06 on a per residue basis.**
(PDF)

Figure S3 **Phylogenetic tree of the distribution of the AFV1p06-like ZNF fold in** ***Eukarya*** **and** ***Archaea*** **.**
(PDF)
