# Supplementary material for: Solution Structure of an Archaeal DNA Binding Protein with an Eukaryotic Zinc Finger Fold
Source: PLoS One. 2013 Jan 9;8(1):e52908. doi: 10.1371/journal.pone.0052908 (PMC3541406; doi:10.1371/journal.pone.0052908)
Supplement: Figure S1 — Assigned 1H-15N HSQC spectrum of AFV1p06. (PDF) [file pone.0052908.s001.pdf]

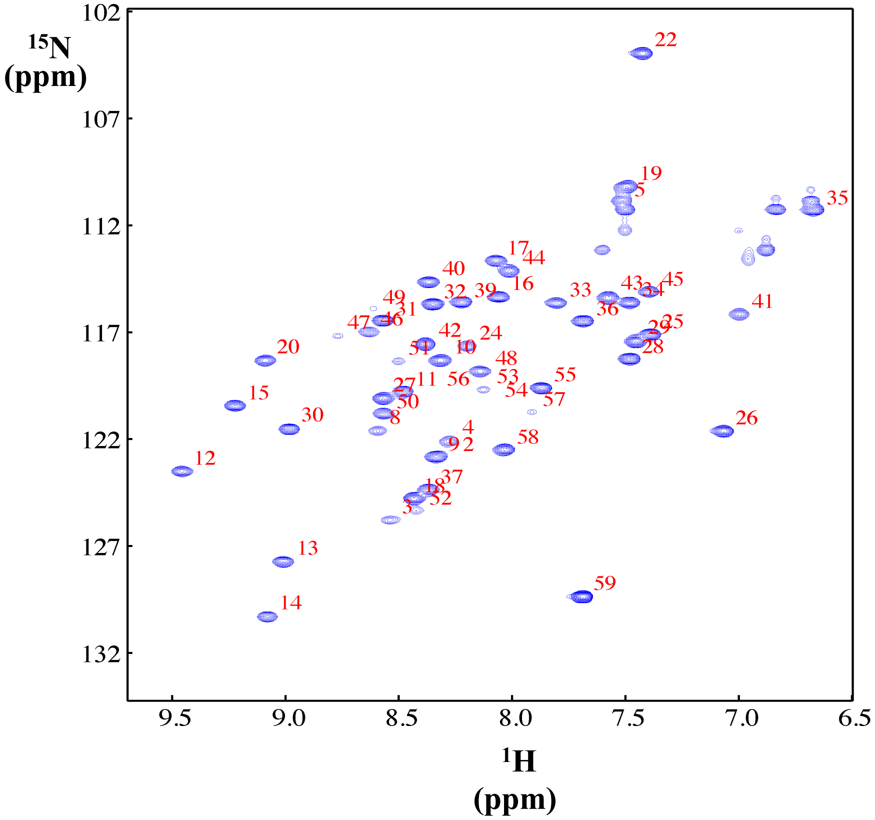

Figure S1: Assigned ^{1}H-^{15}N HSQC spectrum of AFV1p06 obtained at 25 °C in buffer E. Assignments are indicated in red. Assignments of side-chain amide groups are not shown.
