# Supplementary material for: Solution Structure of an Archaeal DNA Binding Protein with an Eukaryotic Zinc Finger Fold
Source: PLoS One. 2013 Jan 9;8(1):e52908. doi: 10.1371/journal.pone.0052908 (PMC3541406; doi:10.1371/journal.pone.0052908)
Supplement: Figure S2 — Structural (number of nOe-derived distance constraints and structure backbone RMSD) and dynamics (amide 15N transverse relaxation rate) data of AFV1p06 on a per residue basis. (PDF) [file pone.0052908.s002.pdf]

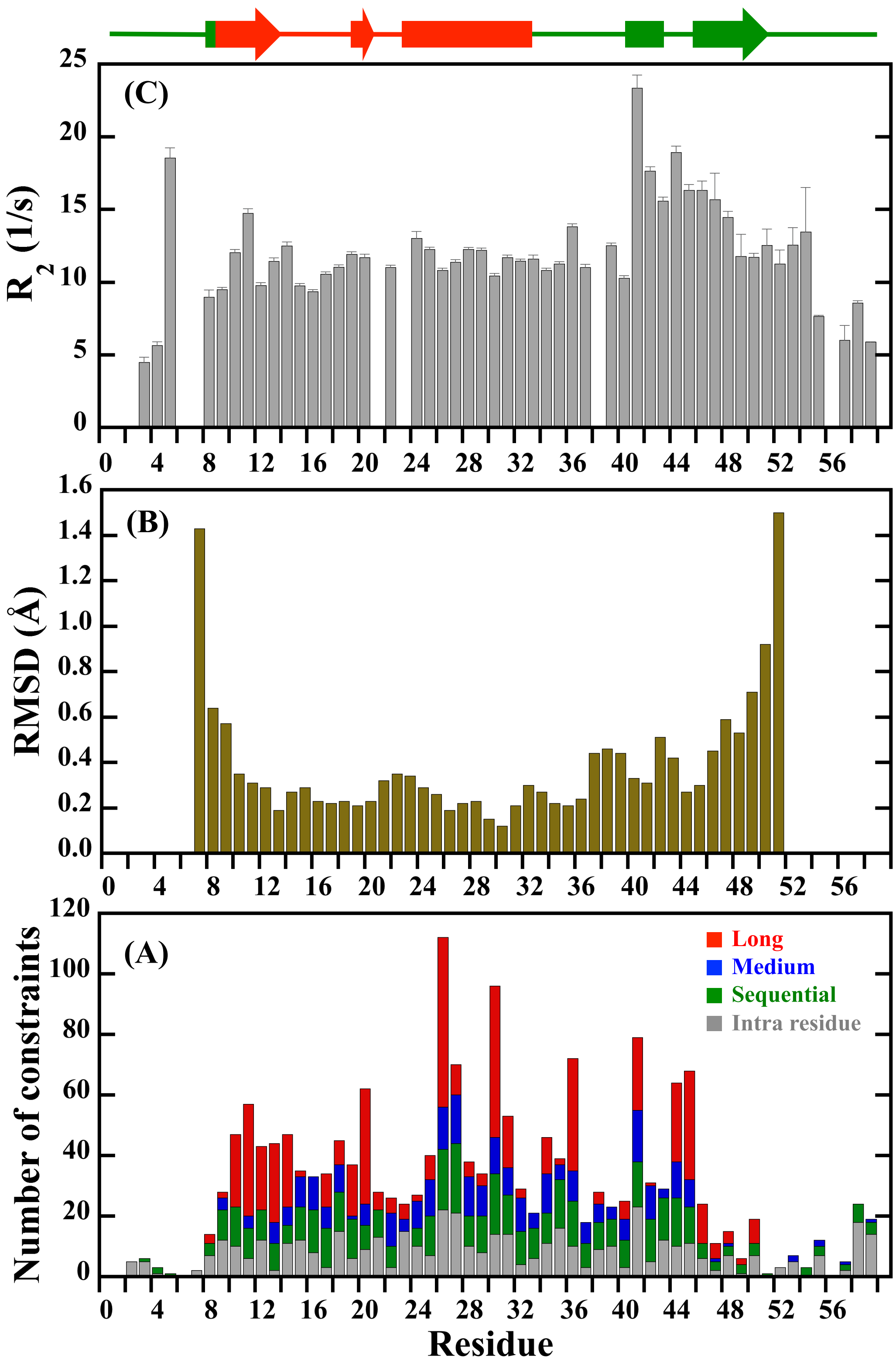

Figure S2: Number of nOe-derived distance constraints per residue used for structure calculations **(A)**; mean pairwise root mean square deviation (RMSD) of backbone atoms (N,C,O,CA) of the structure ensemble **(B)**; amide ^{15}N transverse relaxation rate (R_2) **(C)**. The intra-residue, sequential, medium and long range distance constraints are colour coded in (A). In (B), the RMSD of residues 1-6 and 52-59, which are disordered, are not shown. The secondary structure is displayed on the top of the figure, with helices represented as rectangles and strands as arrows. The ZNF region is displayed in red and the rest of the protein in green.
