# Supplementary material for: Solution Structure of an Archaeal DNA Binding Protein with an Eukaryotic Zinc Finger Fold
Source: PLoS One. 2013 Jan 9;8(1):e52908. doi: 10.1371/journal.pone.0052908 (PMC3541406; doi:10.1371/journal.pone.0052908)
Supplement: Figure S3 — Phylogenetic tree of the distribution of the AFV1p06-like ZNF fold in Eukarya and Archaea . (PDF) [file pone.0052908.s003.pdf]

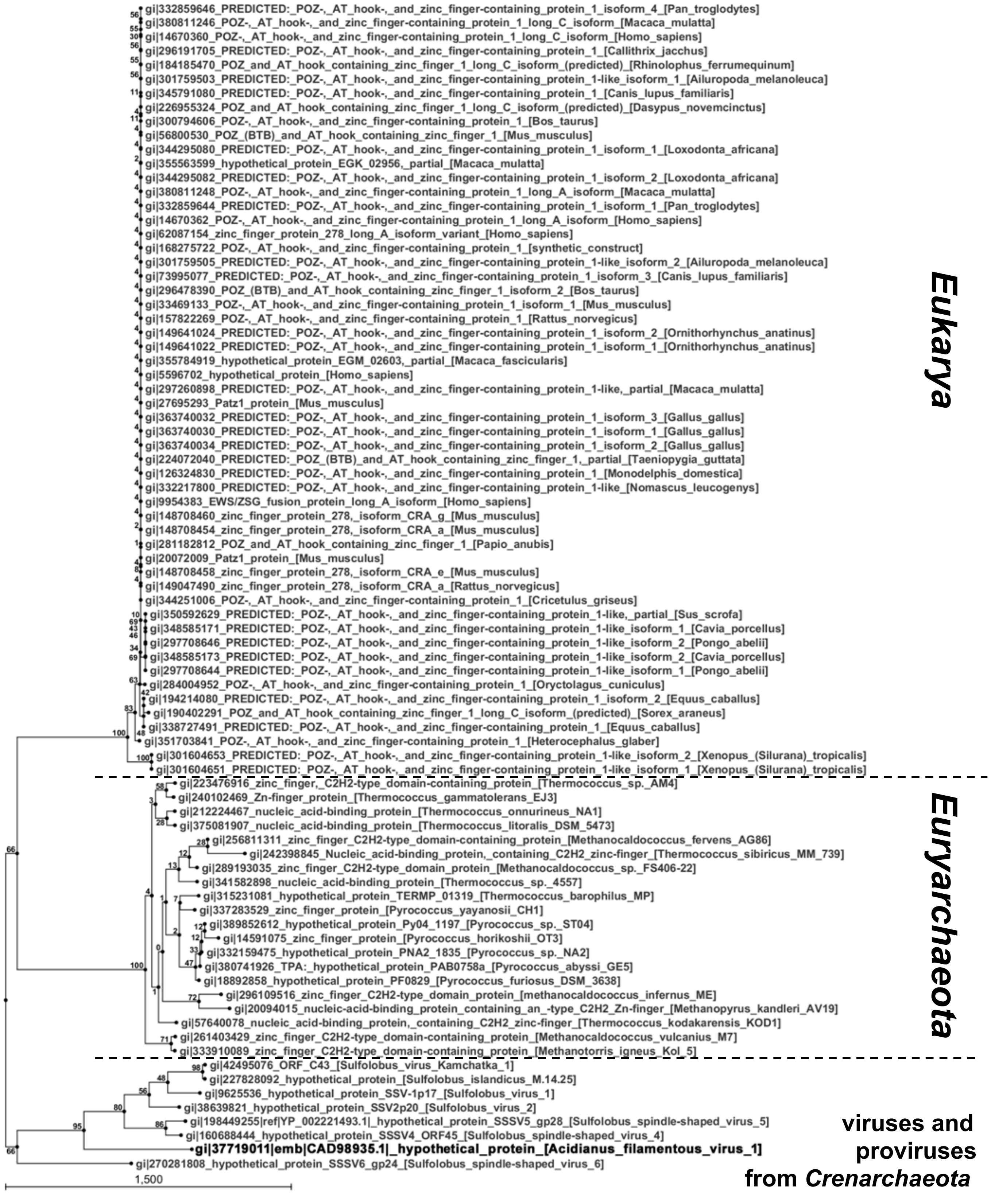

Figure S3: Phylogenetic tree of the distribution of the AFV1p06-like ZNF fold in *Eukarya* and *Archaea*. The tree was built from a psi-blast search of the NCBI database using the alignment of the AFV1p06-like ZFN motif (presented on Fig. 4). Although the separation on two sister groups *Crenarchaea* and *Eukarya/Euryarchaea* was strongly confirmed by the boot strap value, it cannot be excluded that the ZFN proteins found in *Eukarya* and *Euryarchaea* form a single phylogenetic clade.
